# Impact of *BRAF*, *TERT*, and novel mutations on the efficacy of lenvatinib for advanced papillary thyroid cancer: A national genomic database analysis

**DOI:** 10.1038/s41698-026-01371-8

**Published:** 2026-03-18

**Authors:** Yasuyoshi Sato, Naoki Fukuda, Koji Yamamura, Yusuke Ito, Kenya Kobayashi, Yuki Saito, Kousuke Watanabe, Hidenori Kage, Katsutoshi Oda, Shunji Takahashi

**Affiliations:** 1 Department of Clinical Oncology, The University of Tokyo Hospital, Bunkyo-ku, Japan; 2 Department of Medical Oncology, The Cancer Institute Hospital of Japanese Foundation for Cancer Research, Koto-ku, Japan; 3 Departments of Otolaryngology, Head and Neck Surgery, Graduate School of Medicine, The University of Tokyo, Bunkyo-ku, Japan; 4 Department of Clinical Genomics, The University of Tokyo Hospital, Bunkyo-ku, Japan; 5 Department of Respiratory Medicine, Graduate School of Medicine, The University of Tokyo, Bunkyo-ku, Japan

**Keywords:** Biomarkers, Cancer, Genetics, Oncology

## Abstract

Background Lenvatinib is a standard first-line therapy for radioiodine-refractory papillary thyroid carcinoma (PTC). However, the influence of genomic alterations on its efficacy remains unclear. Methods We conducted a nationwide, retrospective study using the Center for Cancer Genomics and Advanced Therapeutics (C-CAT) database in Japan, analyzing 165 patients with PTC who received lenvatinib as first-line treatment. Time to treatment failure (TTF) was compared based on *BRAF* and telomerase reverse transcriptase (*TERT*) promoter mutations and other recurrent genomic alterations. Kaplan–Meier and Cox models were used. Results *BRAF* mutations were present in 79% of patients and *TERT* mutations in 72%. *BRAF* mutation was associated with longer TTF (adjusted hazard ratio [HR]: 0.62, *p* = 0.07). *TERT* mutation alone or in combination with *BRAF* mutation did not affect TTF. Mutations in five genes, *KMT2A*, *MTOR*, *MUTYH*, *CREBBP*, and *RICTOR*, were independently associated with shorter TTF (adjusted HRs 2.04–2.80). These results were supported by variant-level review but did not retain significance after false-discovery-rate adjustment, indicating their exploratory nature. Conclusions Lenvatinib showed substantial efficacy in *BRAF*-mutated PTC, while *TERT* mutations did not predict poor outcomes. The identification of five genes associated with early treatment failure highlights the potential for genomic biomarkers to guide personalized therapy.

## Introduction

Differentiated thyroid carcinoma (DTC), including papillary thyroid carcinoma (PTC), is the most common endocrine malignancy and generally has a favorable prognosis. However, a subset of patients develops radioiodine (RAI)-refractory DTC, which is associated with poor outcomes and limited treatment options. Lenvatinib, an oral multi-tyrosine kinase inhibitor (MKI), has become a standard first-line treatment for RAI-refractory DTC based on the SELECT phase III trial, which demonstrated significantly improved progression-free survival (PFS) compared with placebo^1^. Lenvatinib exerts its anti-tumor effects through selective inhibition of several receptor tyrosine kinases (RTKs) involved in angiogenesis and tumor proliferation, including vascular endothelial growth factor receptors (VEGFR1–3), fibroblast growth factor receptors (FGFR1–4), platelet-derived growth factor receptor alpha (PDGFRα), KIT, and RET^1^.

Concurrently, advances in cancer genomics have enabled molecular subclassification of DTC and identification of prognostically significant mutations. Among these, the *BRAF*^V600E^ mutation, found in 29–69% of PTC cases^2^, is associated with more aggressive histopathological features, resistance to radioiodine therapy, and increased recurrence risk^3,4^. Furthermore, when co-occurring with telomerase reverse transcriptase (*TERT*) promoter mutations, *BRAF* mutations are linked to even poorer prognoses, including reduced overall and disease-free survival^5,6^. Based on these findings, combination therapies targeting the MAPK pathway, specifically, *BRAF* inhibitors with *MEK* inhibitors such as dabrafenib^7^ with trametinib or encorafenib with binimetinib^8^, have emerged as treatment options for *BRAF*-mutant DTC and anaplastic thyroid carcinoma (ATC), supported by phase II trial results. However, current clinical guidelines continue to recommend MKIs such as lenvatinib and sorafenib as first-line treatments for advanced DTC regardless of *BRAF* mutation status, with BRAF/MEK inhibitors reserved for second-line use or specific clinical scenarios^9,10^.

Despite these developments, important clinical questions remain. Notably, the extent to which *BRAF* and *TERT* promoter mutations affect the therapeutic efficacy of lenvatinib in DTC remains unresolved. Although *BRAF* mutations inform the use of targeted MAPK inhibitors, it is unclear whether these mutations or their co-occurrence with *TERT* mutations modulate responsiveness to MKIs such as lenvatinib. Furthermore, while lenvatinib is known to act on multiple RTKs, the role of mutations in these targeted pathways (e.g., VEGFR, FGFR, PDGFRα, RET) as predictive biomarkers of treatment response has not been fully elucidated. To date, no validated genomic markers exist to stratify patients for lenvatinib treatment based on tumor molecular profiles.

To bridge this gap, we leveraged the Center for Cancer Genomics and Advanced Therapeutics (C-CAT) database, a national platform established in Japan to support cancer genomic medicine. C-CAT collects and integrates both clinical data and comprehensive genomic profiling results from patients enrolled in government-approved tumor profiling programs, enabling real-world analyses of genotype–phenotype-treatment relationships in oncology^11,12^. Its use in exploratory biomarker research allows for the systematic identification of clinically relevant genomic alterations.

Lenvatinib was developed for thyroid cancer, even though most PTCs are driven by MAPK pathway mutations (e.g. BRAF, RAS) rather than RTK fusions. The rationale for this stemmed from pre-clinical data showing that angiogenesis is a critical survival pathway in advanced thyroid tumors and from early-phase studies in which broad VEGFR/FGFR blockade produced durable responses when selective MAPK inhibitors were still unavailable^13,14^.

In Japan, comprehensive genomic profiling (CGP) is reimbursed only for patients who lack or who are expected soon to exhaust standard systemic options; consequently, CGP is typically requested at an advanced or refractory stage, and registration in the C-CAT database occurs late in the disease course.

In this study, we aimed to evaluate the impact of *BRAF* and *TERT* promoter mutations on the clinical efficacy of lenvatinib in patients with PTC. Furthermore, using genomic data from the C-CAT database, we sought to identify additional gene mutations that may serve as predictive biomarkers of therapeutic effect. Through this approach, we aimed to clarify the molecular determinants of lenvatinib efficacy and contribute to the optimization of personalized treatment strategies for advanced thyroid cancer.

## Results

### Genomic landscape of the cohort

**Fig. 1:**
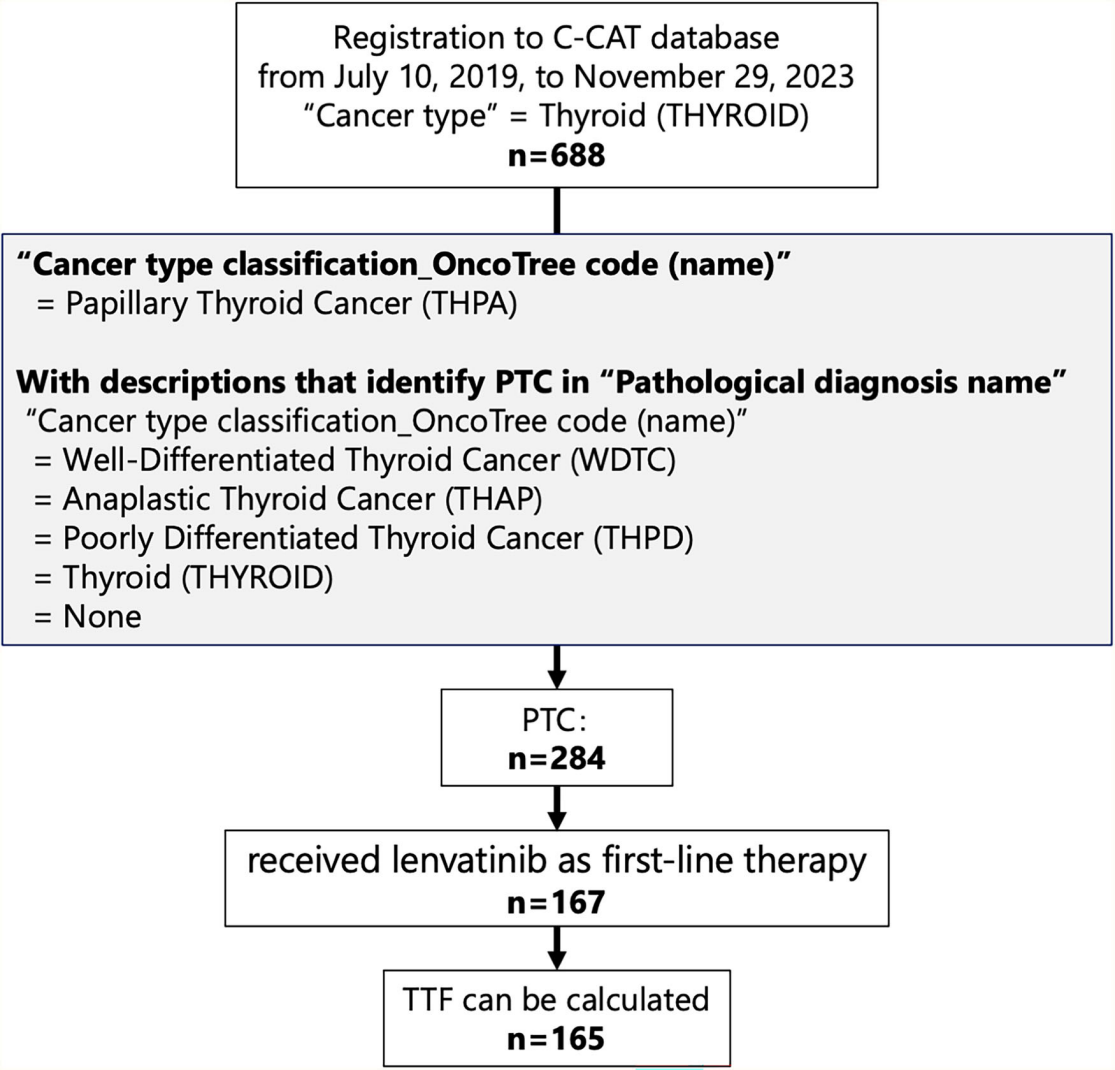
Flow diagram of the study.

Among patients with PTC registered in C-CAT from 10 July 2019 to 29 November 2023, 167 received lenvatinib as first-line therapy. Two patients were excluded because of having no survival data, and 165 patients were enrolled in the study (Fig. 1). The cohort included 72 males (44%) and 93 females (56%) with a median age of 71 years (range 10–86 years). Patient characteristics are presented in Table 1.

**Table 1:** Patient characteristics

Patient characteristics	*n* = 165
*Sex*, *n* *(%)* Male	72 (44)
Age, median [range]	71 [10–86]
*Smoking*	
No	106 (64)
Yes	57 (35)
Unknown	2 (1)
*Alcohol polydipsia*	
No	138 (84)
Yes	18 (11)
Unknown	2 (1)
No data	7 (4)
*ECOG PS at registration, n (%)*	
0	102 (62)
1	59 (36)
2	4 (2)
*Metastatic sites at registration*, *n* *(%)*	
Lung	107 (65)
Lymph nodes	18 (11)
Brain	12 (7)
Bone	11 (7)
Liver	4 (2)
Thyroid	2 (1)
Pleura	2 (1)
Skin	2 (1)
Salivary grand	1 (1)
Pharynx	1 (1)
Muscle	1 (1)
*Genomic profiling test*, *n* *(%)*	
F1CDx	133 (81)
F1Liquid CDx	18 (11)
NCC OncoPanel	14 (8)

*ECOG-PS* Eastern Cooperative Oncology Group performance status, *F1CDx* FoundationOne® CDx, *F1Liquid CDx* FoundationOne® Liquid CDx, *NCC OncoPanel* OncoGuide™ NCC Oncopanel System.

Of 165 patients, *BRAF* mutations were identified in 130 (79%), and 128 of 130 cases (98%) included V600E alterations. *TERT* mutations were observed in 118 patients (72%) (Table 2). Because NCC OncoPanel does not include the *TERT* promoter, we examined panel-specific detection rates. Among the 165 patients, 14 (8.5%) underwent sequencing with NCC OncoPanel, and none showed *TERT* promoter mutations. In contrast, the mutation rate was 84.9% with FoundationOne CDx and 27.8% with F1 Liquid CDx. After excluding the 14 NCC OncoPanel cases, the overall *TERT* mutation frequency was 78.1%.

**Table 2:** Frequency of *BRAF* and *TERT* mutations in C-CAT data (*n* = 165)

Gene	Alteration	*n* = 165
*BRAF*, *n* (%)	V600E	124 (78)
	V600E, I326V	2 (1)
	V600E, D22N	1 (1)
	V600E, G32_A33del	1 (1)
	D22N	1 (1)
	G30D	1 (1)
*TERT*, *n* (%)	c.-124C>T	107 (65)
	c.-146C>T	10 (6)
	c.-79-6_-79-5ins26	1 (1)

### Association of *BRAF* and *TERT* promoter mutations with treatment outcomes

Patients were categorized into four groups according to the presence or absence of *BRAF* and *TERT* mutations: 104 patients had both *BRAF* and *TERT* mutations (hereafter, ‘dual mutation’), 26 patients had *BRAF* but not *TERT* mutations (BRAF alone), 14 patients had *TERT* mutations but no *BRAF* mutations (TERT alone), and 21 patients had neither *BRAF* nor *TERT* mutations (wildtype). Specimen origin was available for all cases. Among the 165 tumors, 51 (30.9%) were derived from primary lesions and 96 (58.2%) from metastatic sites, while 18 (10.9%) were peripheral blood samples.

**Fig. 2:**
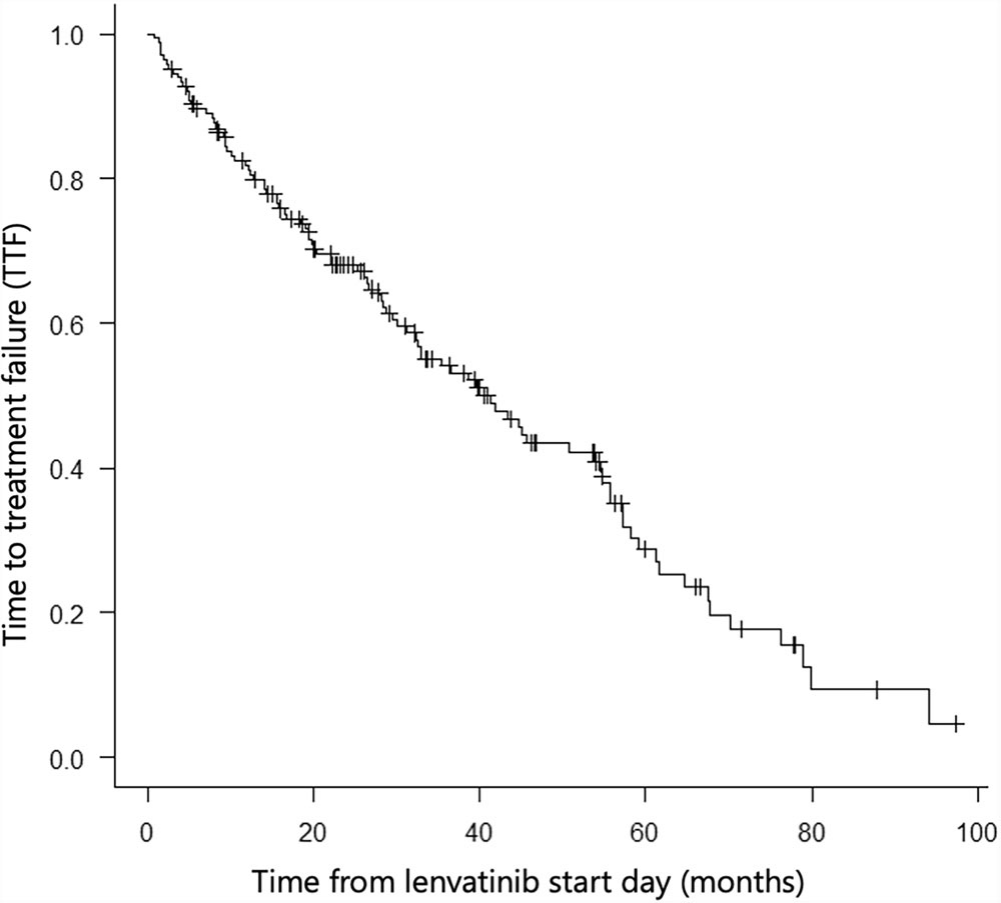
Time to treatment failure (TTF) in the whole patient cohort.

**Fig. 3:**
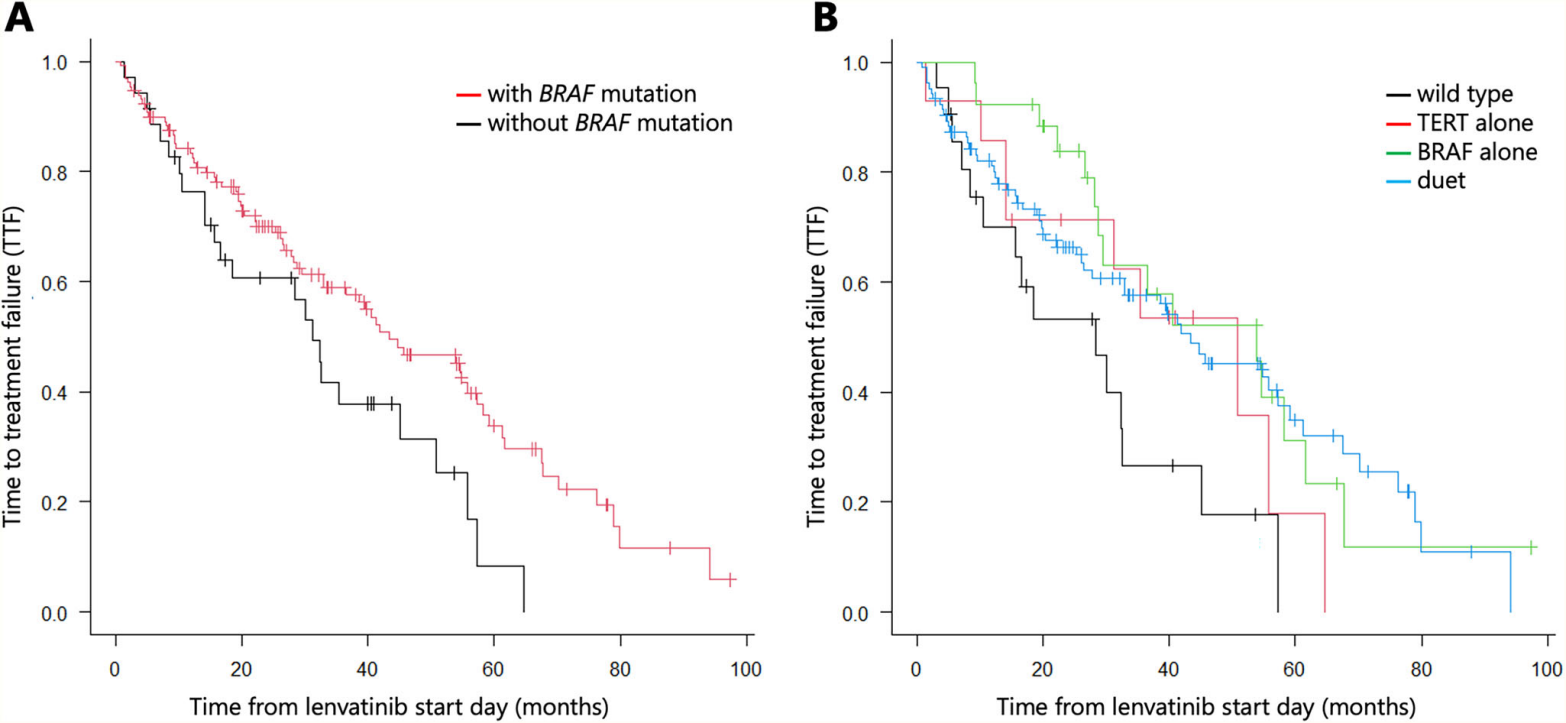
Kaplan–Meier curves for time to treatment failure (TTF). **A** TTF stratifi ed by BRAF mutation status. **B** TTF stratifi ed by combined BRAF and TERT mutation status.

In whole evaluable cases, time to treatment failure (TTF) was 41.3 months (95% confidence interval [CI]: 31.3–53.9) (*n* = 165) (Fig. 2). Among *BRAF* mutations, TTF was 43.3 months (95% CI: 33.0–57.2) in the group with mutations (*n* = 130) and 31.3 months (15.6–50.8) in the group without mutations (*n* = 35), and the difference was statistically significant (*p* = 0.024) (Fig. 3A). When categorized according to the presence or absence of *BRAF* and *TERT* mutations, TTF was 28.4 months (95% CI: 10.4–32.6) in wildtype (*n* = 21), 50.8 months (95% CI: 14.1–NA) in *TERT* alone (*n* = 14), 53.9 months (95% CI: 28.7–61.6) in *BRAF* alone (*n* = 26), and 43.3 months (95% CI: 27.9–57.2) for the double mutation (*n* = 104); the difference was not statistically significant (*p* = 0.058) (Fig. 3B). Although the median TTF was identical (43.3 months) for the overall *BRAF*-mutant cohort and the *BRAF*/*TERT* double-mutant subgroup, this reflects the predominance of double-mutant cases among BRAF-mutant tumors and the substantial proportion of right-censored observations at the time of data lock.

### Additional genomic alterations associated with shorter TTF

The results of univariate and multivariate analyses of factors associated with TTF are shown in Table 3. In the univariate analysis, age ≥65 years versus <65 years showed a hazard ratio (HR) of 0.85 (95% CI: 0.56–1.31, *p* = 0.47). Female sex versus male demonstrated an HR of 0.77 (95% CI: 0.52–1.16, *p* = 0.21), and ECOG performance status ≥1 versus 0 showed an HR of 0.82 (95% CI: 0.54–1.26, *p* = 0.36). Metastatic site other than lung versus lung metastasis had an HR of 1.22 (95% CI: 0.79–1.87, *p* = 0.37), smoking history showed an HR of 1.13 (95% CI: 0.75–1.71, *p* = 0.57), and alcohol polydipsia had an HR of 1.01 (95% CI: 0.55–1.86, *p* = 0.98). Among all these factors, there was no significant association with TTF.

**Table 3:** Univariate and multivariate analyses of factors associated with TTF

Caracteristic	Category	Univariate		Multvariate	
		HR (95% CI)	*p*-value	HR (95% CI)	*p*-value
Age	≥65 vs. <65 years	0.85 (0.56–1.31)	0.47	0.98 (0.62–1.54)	0.93
Sex	Female vs. Male	0.77 (0.52–1.16)	0.21	0.83 (0.55–1.26)	0.38
ECOG PS	≥1 vs. 0	0.82 (0.54–1.26)	0.36		
Metastatic site	Others vs. Lung	1.22 (0.79–1.87)	0.37		
Smoking	Yes vs. No	1.13 (0.75–1.71)	0.57		
Alcohol polydipsia	Yes vs. No	1.01 (0.55–1.86)	0.98		
*BRAF*	Mutant vs. wild	0.58 (0.36–0.94)	0.026	0.62 (0.37–1.04)	0.07
*TERT*	Mutant vs. wild	0.87 (0.56–1.34)	0.52		
*TP53*	Mutant vs. wild	1.31 (0.76–2.24)	0.33		
*PIK3CA*	Mutant vs. wild	1.03 (0.58-1.83)	0.91		
*STK11*	Mutant vs. wild	0.88 (0.46–1.7)	0.7		
*NOTCH3*	Mutant vs. wild	1.21 (0.64–2.28)	0.56		
*LTK*	Mutant vs. wild	1.3 (0.65–2.6)	0.46		
*KMT2D*	Mutant vs. wild	1.55 (0.86–2.79)	0.15		
*ROS1*	Mutant vs. wild	0.72 (0.37–1.39)	0.32		
*ATM*	Mutant vs. wild	1.09 (0.56–2.12)	0.8		
*BRCA2*	Mutant vs. wild	0.97 (0.5–1.87)	0.93		
*NOTCH1*	Mutant vs. wild	1.27 (0.67–2.38)	0.46		
*DNMT3A*	Mutant vs. wild	1.24 (0.66–2.33)	0.51		
*TSC1*	Mutant vs. wild	0.81 (0.41–1.62)	0.55		
*SPEN*	Mutant vs. wild	0.94 (0.47–1.88)	0.87		
*PTCH1*	Mutant vs. wild	1.12 (0.56–2.23)	0.75		
*KMT2A*	Mutant vs. wild	2.01 (1.09–3.71)	0.025	2.04 (1.06–3.93)	0.034
*TET2*	Mutant vs. wild	1.12 (0.56–2.23)	0.75		
*CHEK2*	Mutant vs. wild	0.88 (0.43–1.82)	0.73		
*MTOR*	Mutant vs. wild	2.31 (1.06–5.04)	0.036	2.68 (1.21–5.93)	0.015
*AKT1*	Mutant vs. wild	1.1 (0.48-2.52)	0.82		
*FANCA*	Mutant vs. wild	0.68 (0.28–1.68)	0.4		
*MSH3*	Mutant vs. wild	1.08 (0.47–2.47)	0.86		
*MAP3K1*	Mutant vs. wild	1.06 (0.46–2.42)	0.9		
*MUTYH*	Mutant vs. wild	2.55 (1.27–5.11)	0.0082	2.36 (1.14–4.88)	0.02
*CIC*	Mutant vs. wild	0.94 (0.43–2.03)	0.87		
*NTRK1*	Mutant vs. wild	0.44 (0.18–1.09)	0.07		
*PALB2*	Mutant vs. wild	1.48 (0.68–3.21)	0.33		
*CTNNA1*	Mutant vs. wild	1.26 (0.55–2.89)	0.59		
*TSC2*	Mutant vs. wild	1.48 (0.64–3.41)	0.36		
*PTEN*	Mutant vs. wild	1.69 (0.82–3.49)	0.16		
*CREBBP*	Mutant vs. wild	2.06 (1.03–4.12)	0.041	2.61 (1.27–5.37)	0.0093
*IKBKE*	Mutant vs. wild	1.12 (0.45–2.77)	0.8		
*RBM10*	Mutant vs. wild	1.03 (0.48–2.24)	0.93		
*EP300*	Mutant vs. wild	0.97 (0.36–2.66)	0.96		
*RICTOR*	Mutant vs. wild	3.25 (1.48–7.1)	0.0033	2.8 (1.24–6.35)	0.013
*BCORL1*	Mutant vs. wild	0.88 (0.28–2.79)	0.83		
*PARP3*	Mutant vs. wild	0.85 (0.37–1.94)	0.69		

Among genetic mutations, *BRAF* mutation versus wildtype demonstrated a significant protective effect with an HR of 0.58 (95% CI: 0.36–0.94, *p* = 0.026), while *TERT* mutation showed an HR of 0.87 (95% CI: 0.56–1.34, *p* = 0.52) without significance. We evaluated 38 genes detected in ≥5% of tumors, and most of these alterations, including common drivers such as *TP53*, *PIK3CA*, and *KMT2D*, showed no significant association with TTF (HR range 0.44–1.69). However, five mutations demonstrated significant associations with increased treatment failure risk: *KMT2A* mutation showed an HR of 2.01 (95% CI: 1.09–3.71, *p* = 0.025), *MTOR* mutation had an HR of 2.31 (95% CI: 1.06–5.04, *p* = 0.036), *MUTYH* mutation demonstrated an HR of 2.55 (95% CI: 1.27–5.11, *p* = 0.0082), *CREBBP* mutation showed an HR of 2.06 (95% CI: 1.03–4.12, *p* = 0.041), and *RICTOR* mutation exhibited the highest HR of 3.24 (95% CI: 1.48–7.1, *p* = 0.0033). When adjusted for multiple testing using the Benjamini–Hochberg false discovery rate (FDR), none of these associations met statistical significance (minimum *q* = 0.12; Supplementary Table S1). In the multivariate analysis, variables with *p*-values of <0.05 in the univariate analysis were included along with age and sex for adjustment. Age ≥65 years versus <65 years showed an adjusted HR of 0.98 (95% CI: 0.62–1.54, *p* = 0.93), and female sex versus male sex had an adjusted HR of 0.83 (95% CI: 0.55–1.26, *p* = 0.38), both remaining non-significant. *BRAF* mutation versus wildtype demonstrated a borderline significant protective effect with an adjusted HR of 0.62 (95% CI: 0.37–1.04, *p* = 0.07).

All five mutated genes that showed significance in the univariate analysis maintained their significant associations with treatment failure: *KMT2A* mutation had an adjusted HR of 2.04 (95% CI: 1.06–3.93, *p* = 0.034), *MTOR* mutation showed an adjusted HR of 2.68 (95% CI: 1.21–5.93, *p* = 0.015), *MUTYH* mutation demonstrated an adjusted HR of 2.36 (95% CI: 1.14–4.88, *p* = 0.02), *CREBBP* mutation had an adjusted HR of 2.61 (95% CI: 1.27–5.37, *p* = 0.0093), and *RICTOR* mutation maintained the highest adjusted HR of 2.8 (95% CI: 1.24–6.35, *p* = 0.013). To assess whether specimen origin influenced genomic patterns, we compared mutation frequencies between primary and metastatic tumors. The prevalence of *BRAF* (78.4% vs. 86.5%), *TERT* (68.6% vs. 81.2%), and the five candidate genes showed similar distributions between primary and metastatic specimens (Supplementary Table S2).

To clarify whether the observed associations were driven by pathogenic alterations or by noncanonical calls, we performed a variant-level review of all mutations in the five candidate genes. Across *KMT2A*, *CREBBP*, and *RICTOR*, all detected mutations were noncanonical missense variants without known hotspot or loss-of-function alterations. In contrast, *MTOR* harbored several missense changes previously reported as activating in the literature, whereas *MUTYH* included well-characterized loss-of-function alterations (Supplementary Table S3). These findings indicate substantial variant-level heterogeneity and suggest that associations involving genes dominated by noncanonical missense variants should be interpreted with caution.

Among the 165 patients, 10 (6.1%) harbored *RAS* mutations (*KRAS*, *NRAS*, or *HRAS*). In univariate analysis, *RAS*-mutant versus *RAS*–wild-type tumors showed an HR of 1.07 (95% CI 0.43–2.64, *p* = 0.89). After adjustment for age, sex, *BRAF*, and *TERT* status, *RAS* mutations were likewise not associated with TTF (adjusted HR 0.77, 95% CI 0.29–2.04, *p* = 0.60) (Supplementary Table S4).

We performed an internal split-sample validation to assess the robustness of the five-gene signal. The cohort was randomly divided (2:1) into training (*n* = 110) and test (*n* = 55) sets. We constructed a composite variable (“any of the five genes mutated”) and evaluated its association with TTF. In the training set, the composite variable was associated with shorter TTF (HR 2.82, 95% CI 1.68–4.75, *p* < 0.001). In the independent test set, the hazard ratio remained consistent in direction and magnitude (HR 3.84, 95% CI 1.63–9.05, *p* = 0.002) (Supplementary Table S5). These results indicate that the adverse association of the five-gene panel with TTF was reproducible within this cohort.

## Discussion

In this real-world analysis using the C-CAT national genomic database, we evaluated the clinical efficacy of lenvatinib as first-line therapy and its association with genomic alterations in 165 patients with PTC. The median TTF was 41.3 months, which was substantially longer than the median PFS of 18.3 months reported in the phase III SELECT trial^1^. Clinical background factors such as age, sex, ECOG PS, and metastatic sites were not significantly associated with treatment duration. Among genetic variables, *BRAF* mutations were significantly associated with prolonged TTF (HR 0.58), with a borderline effect in the multivariate analysis. Conversely, mutations in five genes, *KMT2A*, *MTOR*, *MUTYH*, *CREBBP*, and *RICTOR*, were significantly associated with a two- to three-fold increased risk of treatment failure. These findings suggest that molecular profiles, rather than clinical factors, play a critical role in determining the efficacy of lenvatinib in PTC.

It is important to recognize several structural features of CGP implementation in Japan that shape how real-world treatment duration is captured in the C-CAT registry. Because insurance coverage restricts CGP to patients who have exhausted, or are expected soon to exhaust, standard treatment options, genomic testing is typically performed relatively late in the disease course. As a result, patients must survive long enough without early rapid progression and remain physically fit enough to undergo CGP procedures, which inherently enriches the registry for individuals who are both “late-entrants” and “fit-survivors.” These selection processes plausibly contribute to the longer TTF observed in real-world practice compared with the SELECT trial. Additionally, in routine practice, lenvatinib failure is not declared when temporary dose interruptions are followed by successful re‑escalation and renewed tumor control, whereas any progression during protocol‑mandated holds is counted as failure in SELECT; this real‑world approach, effective in 43% of radioiodine‑refractory DTC cases^27^, plausibly extends TTF beyond trial‑defined PFS. For these reasons, all hazard ratios in this study were calculated using TTF, which represents the most consistently ascertainable follow-up metric available within the constraints of C-CAT, where uniform PFS or OS could not be reliably captured.

The observed frequencies of *BRAF* (79%) and *TERT* promoter (72%) mutations in our cohort were higher than those reported in unselected PTC series^2^, likely reflecting the advanced disease characteristics of C-CAT registrants. As *TERT* alterations accumulate with tumor dedifferentiation and metastatic progression^15^, enrichment in this advanced cohort is biologically plausible. These frequencies also align with prior C-CAT studies (*BRAF* 75%, *TERT* 71%)^16^. Importantly, NCC OncoPanel lacks *TERT* promoter coverage, but exclusion of these cases did not materially alter mutation frequencies, supporting the robustness of our mutation distribution estimates and indicating that incomplete *TERT* coverage had minimal impact on downstream analyses.

Although *BRAF*^*V*600E^ mutations have been reported to be associated with RAI resistance, poor prognosis, and tumor recurrence in PTC^5,6^, our results showed longer TTF in *BRAF*-mutant cases. This is consistent with the SELECT exploratory analysis, which suggested maintained PFS benefit regardless of BRAF status^17^, and with real-world US data showing similar lenvatinib activity across *BRAF* subgroups^18^. One explanation is that *BRAF*^V600E^-mutant PTC exhibits higher VEGF expression and angiogenic dependency^19,20^, potentially enhancing sensitivity to VEGFR/FGFR inhibition. However, *BRAF*/MEK inhibitors were approved for DTC in Japan only in November 2023, and the patients in our study were registered between July 2019 and November 2023. Therefore, their impact on TTF in this cohort is likely negligible. Conversely, *BRAF*-wild-type cases may include *RAS*-like tumors with poorer intrinsic prognosis, which prompted us to examine *RAS* mutations as a separate subgroup^21^.

*RAS* mutations represent an alternative oncogenic driver in differentiated thyroid carcinoma and are associated with a “*RAS*-like” transcriptional program as well as adverse biological features in some series^21,22^. Consistent with prior reports that many *BRAF*-wild-type DTCs are *RAS*-driven, 7 of 34 *BRAF*-wild-type tumors in our cohort harbored *RAS* mutations. In our cohort, however, *RAS*-mutant tumors were infrequent (*n* = 10) and showed no association with treatment duration. These findings suggest that, within advanced lenvatinib-treated PTC, *RAS* status alone is unlikely to be a major determinant of TTF, although the small number of *RAS*-mutant cases warrants cautious interpretation.

While prior studies have reported that *BRAF* and *TERT* promoter mutations correlate with decreased overall survival^5,6^, notably, these earlier analyses were conducted almost exclusively in postoperative cohorts and therefore reflect a higher risk of recurrence after surgery rather than prognosis in established advanced disease; their conclusions are not directly transferable to the treatment‑refractory setting examined here. Our results showed no significant impact of *TERT* mutation alone or in combination with *BRAF* mutations on TTF. Collectively, our findings suggest that the presence of *BRAF* and/or *TERT* mutations should not preclude lenvatinib as a first-line therapy option in advanced PTC.

**Fig. 4:**
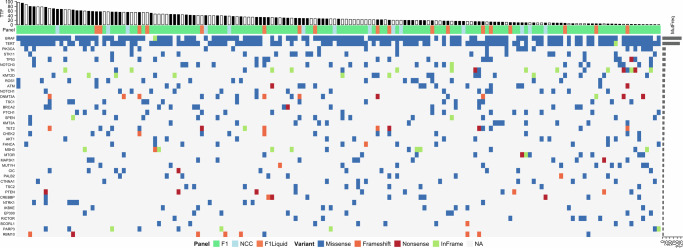
Oncoprint of the lenvatinib-treated C-CAT PTC cohort (*n* = 165). Columns represent individual tumors ordered left-to-right by decreasing time-to-treatment-failure (TTF). Top barplot (TTF). Bars show the TTF in months; black = event, white = censored. Panel row. Color indicates the comprehensive genomic-profiling assay used: FoundationOne CDx tissue (F1, green), FoundationOne Liquid CDx (F1 Liquid, orange), or NCC OncoPanel (NCC, light blue). Mutation matrix. Squares depict non-synonymous variants in the 38 genes mutated in ≥5% of tumors; variant classes are color-coded (missense, frameshift, nonsense, in-frame indel, NA no alteration). Right barplot (MutFreq). Absolute mutation counts for each gene.

Our study identified five gene mutations associated with shorter TTF for lenvatinib in PTC. *KMT2A* and *CREBBP* encode chromatin-modifying enzymes that regulate angiogenesis and tumor progression through epigenetic mechanisms. In particular, *KMT2D* (a KMT2 family member) is upregulated in PTC and promotes tumor cell proliferation and invasion via activation of the NCOA6/THRB signaling axis^23^. Furthermore, *CREBBP* has been shown to acetylate *KMT2D*, with both proteins acting cooperatively at enhancer regions, suggesting a role in aggressive tumor behavior and potential treatment resistance^24^. *MTOR* and *RICTOR* are central components of the mTORC1 and mTORC2 complexes, respectively, and play crucial roles in regulating tumor metabolism and survival. Notably, SIN1, an essential adaptor protein for mTORC2 function, is overexpressed in aggressive PTC and correlates with enhanced *AKT* phosphorylation, indicating activation of the mTORC2–AKT pathway as a potential mechanism of lenvatinib resistance^25^. To clarify their genomic context, the 38-gene Oncoprint (Fig. 4) shows that these mutations appear sporadically and rarely co-occur, suggesting that they represent independent biological subsets rather than a single convergent pathway.

*MUTYH* encodes a DNA glycosylase involved in base excision repair and genomic stability. Recent genomic profiling studies have identified somatic and germline *MUTYH* mutations in ATC, with some cases lacking *BRAF* mutations, suggesting an independent oncogenic role^26^. Because ATC can arise from PTC, and dedifferentiation is a known mechanism of lenvatinib resistance^27^, *MUTYH* mutations may act as early indicators of treatment resistance. Additionally, thyroid carcinoma has been reported as an initial manifestation in patients with MUTYH-associated polyposis, further supporting a tumorigenic role in thyroid tissue^28^.

Although exploratory, the biological pathways implicated by MTOR/RICTOR, KMT2A/CREBBP, and MUTYH raise the possibility that alterations in downstream signaling, epigenetic regulation, or DNA-repair programs may modulate lenvatinib sensitivity. These considerations remain hypothetical, but they outline mechanistic directions that could be assessed in future preclinical studies to clarify whether specific molecular contexts contribute to early treatment failure. Their therapeutic relevance, if any, remains to be determined.

To assess robustness, we performed an internal 1:1 split-sample validation. The composite presence of any of the five mutations remained associated with shorter TTF in both the training (HR 2.82) and test (HR 3.84) cohorts, supporting the reproducibility of the signal despite its exploratory nature. Our study is the first to clinically demonstrate that these mutations may also affect lenvatinib efficacy, reinforcing the rationale for personalized treatment strategies such as combination therapy with mTOR inhibitors or epigenetic modulators in mutation-positive cases.

Because the five genes included biologically plausible pathogenic variants as well as numerous noncanonical missense alterations, we examined variant-level evidence. All mutations in KMT2A, CREBBP, and RICTOR were noncanonical missense variants lacking validated functional consequences, whereas MTOR and MUTYH included variants consistent with known activating or loss-of-function biology. Thus, some associations may reflect variant-level uncertainty rather than definitive biological effects, underscoring the importance of independent validation. Finally, we applied a Benjamini–Hochberg FDR correction across all 38 genes detected in ≥5% of tumors. No associations remained significant after adjustment, indicating that the findings should be considered hypothesis-generating rather than definitive.

Current guidelines do not recommend specific molecular biomarkers to guide first-line lenvatinib use^9,10^. Our findings challenge this view by demonstrating that *BRAF* and *TERT* mutations do not predict poor response to lenvatinib, while alterations in *MTOR*, *MUTYH*, and related chromatin/mTOR pathway genes may identify patients at higher risk of early treatment failure. The C-CAT platform provides a unique opportunity for nationwide validation of genomic biomarkers and efficient clinical trial matching^12^.

This study has several limitations. First, CGP testing in Japan is typically performed late in the disease course and only in patients sufficiently fit to undergo testing; together with the retrospective study design, this introduces late-entry and healthy survivor biases as well as potential unmeasured confounding, limiting the generalizability of our findings to the broader population of patients receiving first-line lenvatinib. Second, although progression-free survival is the standard efficacy endpoint in clinical trials, TTF was selected in this study because radiographic progression dates and RECIST-based assessments are not uniformly captured in C-CAT, whereas treatment initiation and discontinuation dates are reliably recorded. As a result, TTF is influenced not only by tumor progression but also by clinical decision-making, institutional practice, and adverse events, and may not directly reflect biological efficacy. Moreover, in this real-world dataset, long-term treatment continuation was frequently right censored at the time of data lock, and median TTF estimates should therefore be interpreted as reflecting early treatment failure rather than long-term treatment durability. Third, heterogeneity in genomic profiling platforms may have affected mutation detection, as the NCC OncoPanel does not cover the *TERT* promoter region, potentially leading to underestimation of *TERT* mutations in a subset of cases, although sensitivity analyses suggested minimal impact. Fourth, the “metastatic site” field in C-CAT reflects the treating physician’s judgment rather than the AJCC TNM M1 definition; therefore, entries such as “thyroid” may represent local recurrence rather than true distant metastasis. However, this limits comparability with staging-based datasets. Fifth, specimen heterogeneity could influence genomic profiles; however, mutation frequencies in primary versus metastatic tumors were highly concordant. Finally, retention of VUS was necessary to avoid discarding potentially meaningful emerging variants, but may introduce nondifferential misclassification; findings based on noncanonical missense variants (e.g., *RICTOR*) should therefore be interpreted conservatively.

In conclusion, the present nationwide real‑world study leveraging the C‑CAT database aimed to clarify how *BRAF* and *TERT* promoter mutations affect the efficacy of first‑line lenvatinib in PTC and to identify additional genomic biomarkers. BRAF‑mutant tumors experienced longer TTF, while *TERT* mutations, alone or co‑occurring with *BRAF* mutations, had no adverse impact. Most importantly, we identified previously underappreciated alterations in five genes, *KMT2A*, *MTOR*, *MUTYH*, *CREBBP*, and *RICTOR*, that conferred a two‑ to three‑fold increase in treatment‑failure risk. Together, these findings highlight that lenvatinib remains a highly effective first‑line option even in BRAF‑ or TERT‑mutated PTC, and indicate new precision strategies by refining risk stratification and guiding combination therapies to overcome its resistance.

## Methods

### Study design and data source

We conducted this retrospective observational study using data from the C-CAT database. This database contains comprehensive clinical information including: age, sex, cancer type, pathological diagnosis, smoking and alcohol drinking history, Eastern Cooperative Oncology Group Performance Status (ECOG PS), and metastatic organs; treatment information encompassing detailed chemotherapy regimen data with start and end dates for each regimen, antitumor response prior to chemotherapy, and severe adverse events (AEs) prior to chemotherapy; clinical trial information based on cancer gene panel test results; and genomic information including types of detected abnormal genes, mutation types, frequency of genetic mutations, and clinical significance.

### Patient selection and eligibility criteria

The study included cases registered in C-CAT between 10 July 2019 and 29 November 2023, specifically targeting PTC patients who received lenvatinib as first-line treatment. PTC cases were extracted from cases with “Cancer type” designated as thyroid (THYROID) using two approaches: cases with “Cancer type classification_OncoTree code (name)” categorized as Papillary Thyroid Cancer (THPA), and cases with “Cancer type classification_OncoTree code (name)” classified as Well-Differentiated Thyroid Cancer (WDTC), Anaplastic Thyroid Cancer (THAP), Poorly Differentiated Thyroid Cancer (THPD), Thyroid (THYROID), or None, provided that the “Pathological diagnosis name” field documented PTC. From the extracted PTC cases, patients who received lenvatinib as first-line treatment were further identified, with exclusion of cases lacking sufficient data regarding the start and end dates of first-line treatment^29,30^.

### Genomic profiling and mutation definition

In this study, every variant listed in the vendor reports was considered positive irrespective of its exact variant allele frequency. Variants of uncertain significance (VUS) reported by the vendor were retained; no manual re-annotation was performed.

### Outcome definition

The primary endpoints included characterization of genetic mutations and their frequencies, with the extraction of gene mutations observed in 5% or more of the cases, and assessment of time to TTF for lenvatinib therapy. TTF was defined as the interval from lenvatinib initiation to treatment discontinuation or last follow-up at the time of the most recent C-CAT data lock.

### Statistical analysis

Comparative analysis of TTF was conducted based on the presence or absence of *BRAF* gene mutations and *TERT* gene promoter region mutations. Univariate analysis was performed using TTF as the outcome variable and the following factors as variables: age (≥65 vs. <65 years), sex (female vs. male), ECOG performance status (≥1 vs. 0), primary metastatic organ site (others vs. lung), smoking history (yes vs. no), alcohol polydipsia (yes vs. no), and genetic mutations observed in 5% or more of cases (mutant vs. wildtype). Multivariate analysis was subsequently conducted using TTF as the outcome variable and factors with *p*-values < 0.05 from the univariate analysis, along with age and sex, as variables.

In addition to these prespecified analyses, several exploratory analyses were performed to address reviewer concerns. First, a *RAS*-mutant subgroup analysis (*KRAS*, *NRAS*, *HRAS*) was conducted using Cox models. Second, to evaluate the impact of panel-dependent *TERT* promoter detectability, a sensitivity analysis excluding cases sequenced by NCC OncoPanel (which lacks *TERT* coverage) was performed. Third, mutation frequencies were compared between primary and metastatic tumor specimens. Fourth, multiple hypothesis testing across genes detected in ≥5% of cases was addressed using the Benjamini–Hochberg FDR correction. Fifth, to assess robustness, an internal 1:1 split-sample validation was performed by randomly dividing the cohort into training and test sets and repeating the multivariate Cox analysis. Finally, variant-level features were evaluated by classifying alterations into canonical hotspot mutations, truncating variants, and noncanonical missense variants.

TTF was evaluated using Kaplan–Meier curves, with intergroup comparisons performed using the log-rank test. Univariate and multivariate analyses for survival outcomes were conducted using Cox proportional hazards regression. All time-to-event analyses, using both univariate and multivariable Cox models, were performed with TTF as the endpoint because it incorporates the longest available follow-up for each patient and minimizes informative censoring.

All statistical analyses were performed using EZR (Saitama Medical Center, Jichi Medical University, Saitama, Japan), a graphical user interface for R^31^, and R version 4.4.0 (The R Foundation for Statistical Computing, Vienna, Austria).

### Ethics approval

This research was conducted in accordance with the Declaration of Helsinki, and the study protocol received approval from both the Ethics Committee of the Graduate School of Medicine and the Faculty of Medicine, The University of Tokyo (# 2021341G) and the C-CAT Data Utilization Review Board (# CDU2022-026N).

## Supplementary information

Supplementary Information

## Data Availability

The datasets generated and/or analyzed during the current study are not publicly available due to privacy and institutional policy restrictions, but are available from the corresponding author upon reasonable request.

## Code availability

No custom code was used for the analyses in this study. Statistical analyses were performed using standard functions in R. Therefore, code availability is not applicable.
