# Supplementary material for: Impact of BRAF, TERT, and novel mutations on the efficacy of lenvatinib for advanced papillary thyroid cancer: A national genomic database analysis
Source: NPJ Precis Oncol. 2026 Mar 18;10:176. doi: 10.1038/s41698-026-01371-8 (PMC13149585; doi:10.1038/s41698-026-01371-8)
Supplement: Supplementary file 1 — Supplementary Information [file 41698_2026_1371_MOESM1_ESM.pdf]

Supplementary Table S1. Exploratory univariate Cox analyses for 38 genes mutated in ≥5% of tumors, with FDR correction

Gene	HR	95% CI	p-value	q-value (FDR)	Interpretation
RICTOR	3.25	1.48–7.11	0.0033	0.12	Nominal (NS after FDR)
MUTYH	2.55	1.28–5.11	0.0082	0.15	Nominal (NS after FDR)
BRAF	0.58	0.36–0.94	0.026	0.24	Nominal (NS after FDR)
KMT2A	2.01	1.09–3.71	0.025	0.24	Nominal (NS after FDR)
MTOR	2.31	1.06–5.05	0.036	0.25	Nominal (NS after FDR)
CREBBP	2.06	1.03–4.12	0.041	0.25	Nominal (NS after FDR)
NTRK1	0.44	0.18–1.08	0.074	0.39	NS
KMT2D	1.55	0.86–2.79	0.145	0.65	NS
PTEN	1.69	0.82–3.49	0.158	0.65	NS
TERT	0.87	0.56–1.34	0.519	0.96	NS
TP53	1.31	0.76–2.24	0.333	0.96	NS
PIK3CA	1.03	0.58–1.83	0.912	0.96	NS
STK11	0.88	0.45–1.70	0.701	0.96	NS
NOTCH3	1.21	0.64–2.27	0.560	0.96	NS
LTK	1.30	0.65–2.59	0.458	0.96	NS
ROS1	0.72	0.37–1.39	0.322	0.96	NS
ATM	1.09	0.56–2.12	0.802	0.96	NS
BRCA2	0.97	0.50–1.87	0.927	0.96	NS
NOTCH1	1.27	0.67–2.38	0.463	0.96	NS
DNMT3A	1.24	0.66–2.33	0.510	0.96	NS
TSC1	0.81	0.41–1.62	0.551	0.96	NS
SPEN	0.94	0.47–1.88	0.867	0.96	NS
TET2	1.12	0.56–2.23	0.753	0.96	NS
CHEK2	0.88	0.43–1.82	0.732	0.96	NS
AKT1	1.10	0.48–2.52	0.822	0.96	NS
FANCA	0.68	0.28–1.68	0.402	0.96	NS
MSH3	1.08	0.47–2.47	0.860	0.96	NS
MAP3K1	1.06	0.46–2.42	0.899	0.96	NS
CIC	0.94	0.43–2.03	0.874	0.96	NS
PALB2	1.48	0.68–3.21	0.325	0.96	NS
CTNNA1	1.26	0.55–2.89	0.587	0.96	NS
TSC2	1.48	0.64–3.41	0.362	0.96	NS
IKBKE	1.12	0.45–2.77	0.804	0.96	NS
RBM10	1.03	0.48–2.24	0.934	0.96	NS

EP300	0.97	0.36–2.66	0.958	0.96	NS
BCORL1	0.88	0.28–2.79	0.826	0.96	NS
PARP3	0.85	0.37–1.94	0.692	0.96	NS

Abbreviations: FDR, false discovery rate; NS, not significant

All analyses were performed using univariate Cox proportional hazards models with LEN_TTF as the time-to-event variable and LEN_TF as the event indicator. P-values were adjusted using the Benjamini–Hochberg false discovery rate (FDR) method.

Supplementary Table S2. Mutation frequencies in primary vs metastatic specimens

Gene	Primary (n=51), n (%)	Metastatic (n=96), n (%)	p-value*
BRAF	40 (78.4)	83 (86.5)	0.24
TERT	35 (68.6)	78 (81.2)	0.10
KMT2A	7 (13.7)	6 (6.2)	0.14
MTOR	3 (5.9)	8 (8.3)	0.75
MUTYH	5 (9.8)	4 (4.2)	0.28
CREBBP	2 (3.9)	7 (7.3)	0.50
RICTOR	3 (5.9)	5 (5.2)	1.00

* Fisher's exact test (exploratory)

Supplementary Table S3. Variant-level characteristics of mutations in the five genes associated with shorter TTF

Gene	No. of patients	No. of variants	Classification summary	Notes
KMT2A	14	14	All noncanonical missense variants	No known hotspots or truncating variants observed
CREBBP	10	10	All noncanonical missense variants	No frameshift/nonsense variants
RICTOR	9	9	All noncanonical missense variants	Functional relevance uncertain
MTOR	13	13	Mix of reported activating missense variants and noncanonical missense variants	Includes variants consistent with mTOR pathway activation
MUTYH	12	12	Mix of known loss-of-function variants and noncanonical missense variants	Several well-characterized LoF variants identified

Abbreviations: Lof, loss of function

Supplementary Table S4. Association of RAS mutations with TTF

Model	HR	95% CI	p-value
**Univariate**	1.07	0.43–2.64	0.886
**Multivariate***	0.77	0.29–2.04	0.599

*Adjusted for age, sex, BRAF, and TERT status.

Supplementary Table S5. Internal split-sample validation of the five-gene composite variable

Subset	n	HR	95% CI	p-value
**Training set (2/3 split)**	110	2.82	1.68 – 4.75	<0.001
**Test set (1/3 split)**	55	3.84	1.63 – 9.05	0.0021

Abbreviations: HR, Hazard ratio

Note: Composite variable was defined as presence of any mutation in *KMT2A*, *MTOR*, *MUTYH*, *CREBBP* or *RICTOR*. Split was performed with a 2:1 random allocation. Cox proportional hazards models were used with TTF as the endpoint.

STROBE Checklist

Section / Item	Recommendation	Where addressed
**Title and abstract**		
1(a)	Indicate the study design in the title or abstract	Abstract (“nationwide retrospective study”)
1(b)	Provide an informative and balanced summary of methods and findings	Abstract
**Introduction**		
2	Explain scientific background and rationale	Introduction
3	State specific objectives	Introduction
**Methods**		
4	Present key elements of study design	Methods (“retrospective observational cohort”)
5	Describe setting, locations, dates, recruitment, follow-up	Methods
6	Eligibility criteria, methods of participant selection	Methods
7	Clearly define outcomes, exposures, predictors, confounders	Methods（TTF, genomic alterations defined）
8	Data sources / measurement methods	Methods（C-CAT, CGP panels）
9	Describe efforts to address potential bias	Methods（multivariable Cox model, variant review）
10	Explain how study size was determined	Methods（all eligible patients included）
11	Explain handling of quantitative variables	Methods
12	Describe all statistical methods	Methods（KM, Cox, FDR）
**Results**		
13	Report participant flow, numbers at each stage	Results
14	Give characteristics of study participants	Table 1
15	Report numbers of outcome events / summary measures	Results（TTF estimates）
16	Main results with unadjusted and adjusted estimates and CIs	Results（HR, CI reported）
17	Other analyses (subgroups, interactions, sensitivity)	Results（variant review, train/test validation）
**Discussion**		

18	Summarise key results	Discussion
19	Discuss limitations	Discussion
20	Provide balanced interpretation	Discussion
21	Discuss generalisability	Discussion
**Other information**		
22	Funding	Acknowledgements ("no external funding")
